# Beyond pigments and perfumes: engineering in the carotenoid and apocarotenoid spectrum, novel enzymes, and synthetic biology strategies

**DOI:** 10.3389/fbioe.2025.1716709

**Published:** 2026-01-15

**Authors:** Baradwaj Ravi Gopal, Zhen Q. Wang

**Affiliations:** Department of Biological Sciences, University at Buffalo, The State University of New York, Buffalo, NY, United States

**Keywords:** apocarotenoids, biosynthesis, carotenoids, metabolic engineering, synthetic biology

## Abstract

Carotenoids and apocarotenoids constitute a structurally and functionally sundry class of isoprenoids whose significance extends from photosynthetic light capture and photoprotection to phytohormone signaling, flavor and aroma formation, and emerging biomedical applications. While recent appraisals have emphasized quantitative advances in microbial production, this mini-review adopts a pathway module-centric perspective. We examine each biosynthetic stage from precursor supply, condensation to geranylgeranyl diphosphate (GGPP), phytoene synthesis, desaturation/isomerization, cyclization, hydroxylation, ketolation, epoxidation, and oxidative cleavage, highlighting novel enzymatic variants, mutagenesis studies, fusion strategies, and compartmentalization approaches that impart metabolic control. Special emphasis is placed on recently discovered and engineered enzymes, as well as synthetic biology tools. This review integrates diverse enzyme sources, host ranges across plants, fungi, algae, yeasts, and bacteria, as well as pathway modularity, to provide an updated review of recent literature. We conclude by outlining future directions that highlight gaps and potential areas for future work. This focused synthesis aims to equip researchers with a hierarchical understanding of the pathways and strategies to advance carotenoid and apocarotenoid biosynthesis.

## 1 Introduction

Carotenoids are one of the most diverse and versatile classes of isoprenoids in nature (Gómez-Sagasti et al., 2023). Beyond their roles in photosynthesis, carotenoids contribute to the vivid pigmentation in diverse organisms and serve as precursors to various bioactive apocarotenoids (Nisar et al., 2015; Sun et al., 2018). This duality of structural pigments and bioactive precursors has made carotenoids a key focus of both basic and applied research.

From a commercial standpoint, carotenoids and their oxidative derivatives occupy an exceptionally broad spectrum beyond pigments and perfumes. In nutrition and pharmaceuticals, β-carotene serves as a provitamin A source (Hermanns et al., 2020), lutein and zeaxanthin support eye health (Mrowicka et al., 2022), and apocarotenoids such as crocetin and bixin exhibit antioxidant and anti-inflammatory properties (Imtiaz et al., 2023). In the cosmetics and food industries, carotenoids such as lycopene and astaxanthin are widely used as natural colorants and anti-aging ingredients (Hermanns et al., 2020). In agriculture, apocarotenoids, such as strigolactones and abscisic acid, function as signaling molecules that regulate plant growth and stress responses (Simkin, 2021; Imtiaz et al., 2023).

To meet rising demand, microbial platforms now enable the scalable and sustainable production of high-value carotenoids, such as lycopene, β-carotene, zeaxanthin, and astaxanthin (Jing et al., 2022; Promdonkoy et al., 2024). These systems offer precise control over metabolic flux, avoid the seasonal and environmental limitations of plant cultivation, and allow cost-effective bioprocessing in industrial fermenters. Additionally, apocarotenoids, such as picrocrocin, strigolactones, and vitamin A (retinol), hold immense industrial relevance and are currently being targeted for microbial production (Varghese et al., 2023).

**FIGURE 1:**
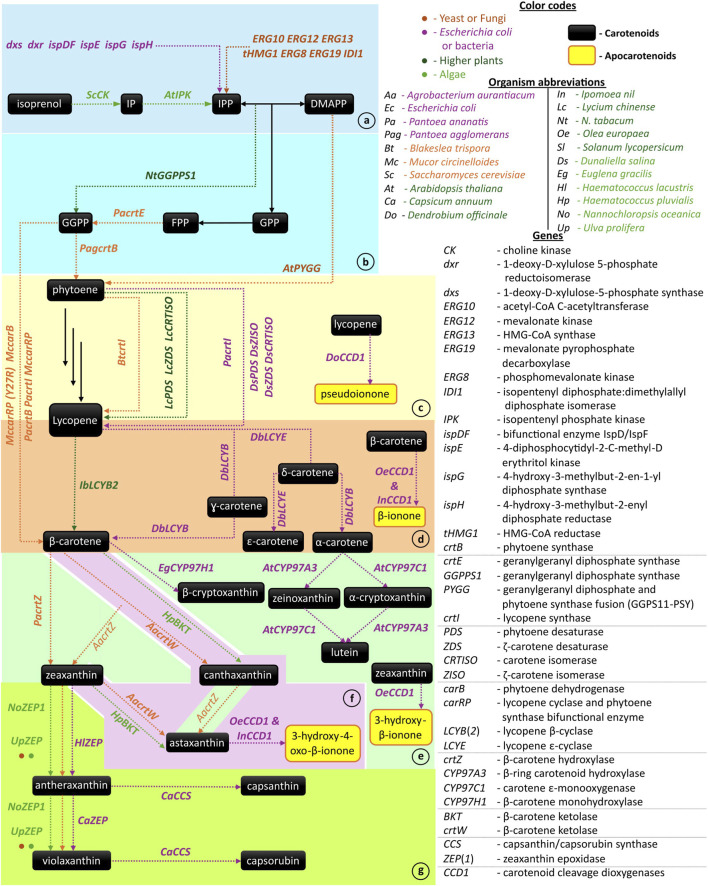
Overview of recent works on carotenoid and apocarotenoid biosynthesis. **(a)** IPP and DMAPP Pathway Engineering, **(b)** Initial Condensation Steps, **(c)** Desaturation and Isomerization, **(d)** Cyclization Reactions, **(e)** Hydroxylation Reactions, **(f)** Ketolation Reactions, **(g)** Epoxidation and Isomerization. (Reactions: Solid black line - native reactions, Dotted colored lines - heterologous reactions. Abbreviations: IP, isopentenyl monophosphate; ISO, isopentenol; IPP, isopentenyl diphosphate; DMAPP, dimethylallyl diphosphate; GPP, geranyl diphosphate; FPP, farnesyl diphosphate; GGPP, geranylgeranyl diphosphate).

Building upon this industrial momentum, it becomes essential to understand the biochemical logic that generates such structural and functional diversity. This diversity stems from a series of biosynthetic transformations, including condensation, desaturation, isomerization, cyclization, hydroxylation, ketolation, and epoxidation. Beyond these modifications, the oxidative cleavage of specific carotenoids by carotenoid cleavage dioxygenases (CCDs) represents a crucial branching point that gives rise to apocarotenoids. Figure 1 summarizes carotenoid and apocarotenoid biosynthetic modules from precursor supply to late-stage tailoring (panels a–g) and distinguishes native reactions (solid black lines) from heterologous/engineered steps (dotted colored lines). Additionally, we review genetic approaches, enzyme sources, host platforms, and outcomes in recent literature, while highlighting emerging trends, persistent bottlenecks, and future directions that bridge fundamental understanding with applied biotechnology, moving beyond pigments and perfumes.

## 2 Engineering strategies by biosynthetic steps

### 2.1 Precursor engineering for carotenoid and apocarotenoid production

Precursor engineering defines the fluxes of carotenoid and apocarotenoid pathways that can be channeled to the final products in any host. Engineering the upstream supply of C5 isoprenoid precursors, isopentenyl pyrophosphate (IPP) and dimethylallyl pyrophosphate (DMAPP), is essential for boosting carotenoid and apocarotenoid yield. The two main biosynthetic routes, the methylerythritol (MEP) and the mevalonate (MVA) pathways, have been extensively engineered in various organisms. The following studies illustrate the control points and how relieving them increases carotenoid precursor levels.

Khana et al. analyzed the bottlenecks of the MEP pathway in the bacterium *Zymomonas mobilis*. They identified 1-deoxy-D-xylulose 5-phosphate synthase (*dxs*) as the first major bottleneck, and 4-hydroxy-3-methylbut-2-enyl diphosphate (HMBDP) synthase (*ispG*) and HMBDP reductase (*ispH*) as the subsequent bottlenecks. Overexpressing *dxs*, *ispG*, and *ispH* alleviated these blocks, while the introduction of a heterologous isoprene synthase provided an effective carbon sink to channel flux (Khana et al., 2023). Similar bottlenecks were identified in the *Escherichia coli* MEP pathway. Raghavan et al. showed that *dxs*, *ispG*, and *ispH* constrain flux through the MEP pathway. Chromosomal integration of the whole MEP pathway genes significantly increased the IPP/DMAPP supply compared with episomal expression (Raghavan et al., 2024).

Extensive MVA pathway engineering has been conducted in *Saccharomyces cerevisiae* in recent years. Mukherjee et al. targeted the MVA pathway to both the cytosol and peroxisomes, resulting in a 94-fold increase in the titer of monoterpene geraniol (Mukherjee et al., 2022). Additionally, they revealed that mevalonate kinase (ERG12) is another bottleneck, along with HMG-CoA reductase (HmgR) and isopentenyl-diphosphate isomerase (IDI). Yanagibashi et al. targeted the mevalonate (MVA) pathway to mitochondria, where the substrate acetyl-CoA is abundant, resulting in a 15-fold increase in IPP and DMAPP. Furthermore, mutants with enlarged mitochondria showed a 1.3-fold increase in IPP/DMAPP and terpenoids, including squalene and β-carotene, which were increased by 2.8- and 1.4-fold, respectively (Yanagibashi et al., 2024).

Beyond the MEP and the MVA pathways, the isopentenol utilization pathway (IUP) presents a robust alternative to boost IPP/DMAPP production. In *Chlamydomonas reinhardtii*, a choline kinase from *S. cerevisiae* (*ScCK*) converts isoprenol to isopentenyl monophosphate (IP), followed by isopentenyl phosphate kinase from *Arabidopsis thaliana* (*AtIPK*) converting IP to IPP. Isoprenol feeding increased IPP 8.6-fold and limonene production 23-fold (Zhao et al., 2022). Kinase-driven phosphorylation of C5 alcohols (isopentenol and prenol) bypasses native regulation to directly supply IPP and DMAPP. Ma et al. (2023) combined IUP with the native MVA pathway in *S. cerevisiae*, creating a universal IPP/DMAPP platform for mono-, di-, and tetraterpene synthesis. Other microbial studies optimized IUP modules, engineered better kinases, and extended the pathway to *E. coli* and non-conventional hosts like *Yarrowia lipolytica* (Ma X. et al., 2022; Zhao et al., 2022; Ma et al., 2023; Pan et al., 2023; Roth and Ward, 2023; Li G. et al., 2024).

Together, these approaches demonstrate that precursor engineering lays the groundwork for subsequent carotenoid pathway optimizations, as summarized in Figure 1a.

### 2.2 Initial condensation steps—GGPP and phytoene synthesis

Iterative condensation of IPP and DMAPP yields C20 geranylgeranyl diphosphate (GGPP); two GGPP molecules then condense to form C40 phytoene, the first committed step in carotenoid biosynthesis. Expressing bacterial GGPP synthase (GGPPS) *crtE* and phytoene synthase *crtB*, along with downstream enzymes, enabled zeaxanthin production in *Yarrowia lipolytica* at 21.98 mg/L (Xie et al., 2021). Additionally, bifunctional enzymes CarRP (phytoene synthase + lycopene cyclase) and phytoene dehydrogenase CarB from the fungus *M. circinelloides* efficiently convert GGPP to lycopene (Xie et al., 2021).

Recent studies identified novel GGPPS enzymes from higher plants *Liriodendron tulipifera* and *Withania somnifera*, oleaginous yeast *Rhodosporidium toruloides*, and bacterium *Elizabethkingia meningoseptica*, expanding the GGPP biosynthesis toolbox (Zhang et al., 2021; Satta et al., 2022; Srivastava et al., 2022; Yang et al., 2022; Song et al., 2023; Adusumilli et al., 2024). A pentuple mutant of *NtGGPPS* from *Nicotiana tabacum* showed enhanced catalytic efficiency and carotenoid output (Dong et al., 2022).

Recent studies also expanded the potential phytoene synthases (PSY) that could be employed for metabolic engineering. In tomato, fruit-specific expression of the autumn olive PSY (*Eut*PSY), combined with the suppression of lycopene ε-cyclase (*Sl*LCYe), enhanced the accumulation of lycopene and β-carotene (Wang et al., 2020). In the green alga *Dunaliella salina*, *Ds*PSY1 was confirmed to be catalytically active, and its stabilization by the Orange (OR) protein boosted carotenoid levels (Liang M. H. et al., 2023). Expression of tea PSY1 (*Cs*PSY1) in carrot callus increased α- and β-carotene to about 400 and 1250 μg/g dry weight, respectively (Li J. W. et al., 2024). In the archaeon *Haloferax volcanii*, the phytoene synthase (HVO-PSY) was identified as the rate-limiting step for bacterioruberin (C50 carotenoid) biosynthesis (Lin et al., 2025). Finally, in *Pyropia yezoensis,* the first *bona fide* phytoene synthase (*Py*PSY) from a red alga was functionally validated (Li C. L. et al., 2024).

Fusion of GGPPS with PSY also demonstrated promise. The GGPS11-PSY chimera (PYGG) in *Arabidopsis thaliana* allowed efficient GGPP channeling to phytoene. Despite a lower turnover rate, this fusion protein showed high substrate utilization and minimal GGPP leakage (Camagna et al., 2019). This strategy may be extendable to other GGPPS and PSY enzymes.

The initial condensation steps determine how effectively the C5 precursor carbons can commit to carotenoid backbones, as depicted in Figure 1b, which connects IPP/DMAPP condensation to phytoene formation.

### 2.3 Desaturation and isomerization—phytoene to lycopene conversion

Desaturation and isomerization of phytoene to lycopene are key bottlenecks in carotenoid biosynthesis. Engineering across diverse hosts has utilized both one-step bacterial or fungal enzymes, such as CrtI, and multi-enzyme plant modules including the phytoene desaturase (PDS), the 15-cis-ζ-carotene isomerase (ZISO), the ζ-carotene desaturase (ZDS), and the carotenoid isomerase (CRTISO).

Notably, CrtI from bacterium *Pantoea ananatis* and red-pigmented yeast *Xanthophyllomyces dendrorhous* enables a streamlined, high-yield conversion of phytoene to lycopene in *E. coli* and *S. cerevisiae,* respectively, when codon-optimized and paired with FAD support (Yoon et al., 2007; Schaub et al., 2012; Hong et al., 2019). Diversified *crtI* homologs across evolutionary clades demonstrated broad variability in desaturation and could be used for bioengineering (Fournié and Truan, 2020). Integrative pathway designs, such as expression of heterologous *Pantoea ananatis crtE, Pantoea agglomerans crtB,* and *Blakeslea trispora crtI*, along with membrane efflux transporters, further enhance yield by relieving product inhibition in *S. cerevisiae* systems, resulting in a lycopene yield of 343.7 mg/L (Huang et al., 2024).

Plant-based strategies emphasize modular control, as demonstrated by overexpressing *Lycium chinense* PDS, ZDS, and CRTISO in *Nicotiana tabacum*, which increased total carotenoid content and enhanced stress tolerance (Li et al., 2020). Other recently identified plant-based phytoene desaturases and isomerases include PDS, ZISO, ZDS, and CRTISO from microalga *Dunaliella salina* (Chen et al., 2023).

In short, desaturation/isomerization is often the first place where tuning redox balance and enzyme expression have a substantial impact on pathway throughput, as shown in Figure 1c, which traces the phytoene-to-lycopene transition catalyzed by CrtI and plant PDS/ZDS modules.

### 2.4 Cyclization reactions—formation of α and β-carotene

Cyclization reactions are key steps in carotenoid biosynthesis, converting lycopene into cyclic carotenoids like β-carotene (two β-ionone rings) and α-carotene (one β- and one α-ionone ring). In plants, LCYB and LCYE catalyze these two reactions, respectively. In microbes, CrtY and CrtL-b form β-carotene, while CrtL-e with CrtL-b/CrtY yields α-carotene (Williamson, 1994; Cunningham Jr and Gantt, 2001). Modulating LCYB and LCYE expression via overexpression, mutagenesis, and promoter tuning regulates carotenoid flux partitioning. Overexpressing *IbLCYB2* in sweet potato boosted β-carotene and stress tolerance (Kang et al., 2018), while bifunctional enzyme *DbLCYB* (lycopene β-cyclase and weak lycopene ε-cyclase activity) from *Dunaliella bardawil* enabled dual cyclization in *E. coli* (Liang et al., 2019). Ma Y. et al. (2022) demonstrated high-yield of β-carotene production in *Yarrowia lipolytica* through expression of the phytoene dehydrogenase (C*arB*) (Velayos et al., 2000a) and bifunctional phytoene synthase and lycopene cyclase *carRP* genes (Velayos et al., 2000b) from *Mucor circinelloides*. It involved overcoming CarRP’s lycopene substrate inhibition by introducing a specific point mutation, Y27R, within its lycopene cyclase domain, alleviating this feedback inhibition. Co-expressing C*arB* and *X. dendrorhous* GGPP synthase *crtE* further improved flux after relieving CarRP inhibition. The engineered strain produced 39.5 g/L β-carotene at a rate of 0.165 g/L/h, which was 1,441-fold higher than that of the parent, highlighting the industrial promise of fungal β-cyclization (Ma Y. et al., 2022).

Recent advances highlight the utility of other fungal systems for lycopene cyclization. In the fungus *Trichoderma reesei*, introduction of the *Mucor circinelloides carB* and the bifunctional *carRP* enabled concurrent cellulase production and β-carotene accumulation (Li et al., 2023). Similarly, in *Yarrowia lipolytica*, expression of *crtE*, *crtB*, *crtI*, and *carRP*, followed by the addition of bacterial β-carotene hydroxylase *crtZ*, achieved efficient β-cyclization and zeaxanthin formation (∼22 mg/L), demonstrating the usefulness of fungal β-cyclases in microbial platforms (Xie et al., 2021).

In *Chlamydomonas reinhardtii*, expressing *crtY* from the bacterium *Pantoea agglomerans* increased β-carotene more than the *Dunaliella salina* lycopene β-cyclase (*DsLCYB1*), showing bacterial cyclases outperform algal ones (Huang et al., 2023). Cross-kingdom enzyme substitution can enhance carotenoid yields in microalgae.

Functionally, this step is the main lever for steering α/β-branch allocation in both plants and engineered microbes, as illustrated in Figure 1d, showing the bifurcation of lycopene into α- and β-carotene via LCYE and LCYB.

### 2.5 Hydroxylation reactions—zeaxanthin, cryptoxanthin, and lutein formation

Hydroxylation increases polarity, converting carotenes into nutritionally and industrially relevant xanthophylls (e.g., zeaxanthin, cryptoxanthins, and lutein) and priming them for further tailoring steps (Cunningham Jr and Gantt, 1998; Cunningham Jr and Gantt, 2001). Key enzymes include non-heme, di-iron type β-carotene hydroxylase (CrtZ/BCH) and cytochrome P450s (CYP97 family), acting on β- or α-ionone rings (Tian et al., 2004; Kim and DellaPenna, 2006; Quinlan et al., 2012). The bacterial *crtZ* gene is preferred for microbial engineering. Enzyme localization, when targeting the bacterial *Pantoea ananatis* CrtZ to peroxisomes or the ER, increased titers, reaching up to 412 mg/L zeaxanthin in *Y. lipolytica* (Soldat et al., 2024). Engineering a β-carotene pathway using *carRP* and *carB* from *Mucor circinelloides*, along with optimized enzyme variants and enhancements to the MVA pathway, increased the supply of the precursor β-carotenoid. The *crtZ* gene from *Pantoea ananatis* enabled zeaxanthin synthesis from β-carotenoid. Further, co-expression of *RFNR1* (ferredoxin–NADP^+^ reductase 1) improved hydroxylation via optimizing electron transfer. This modular strategy achieved a zeaxanthin titer of 775.3 mg/L, among the highest reported in *Yarrowia lipolytica* (Zhang et al., 2023).

CYP97 family members are known to catalyze the hydroxylation of carotenoid ionone rings. Engineering of CYP97H1 and CYP97A3/C1 by strategies such as codon optimization, truncation, cytochrome P450 reductase (CPR) screening, and co-expression enabled hydroxylation of the β-ring of β-carotene and β-/ε-rings of α-carotene, respectively, enabling the production of lutein, zeinoxanthin, α- and β-cryptoxanthin in *E. coli* (Niu et al., 2020; Lautier et al., 2023). Meanwhile, the recently identified CYP97B family enzymes from the diatom Phaeodactylum tricornutum and the red alga *P. umbilicalis* can also hydroxylate β-carotene to zeaxanthin (Yang et al., 2014; Cui et al., 2019). Hydroxylases from bacterium *Chondromyces crocatus* (*CcBCH*), higher plant *Arabidopsis thaliana* (*CYP97A3, CYP97C1*), and algae *Euglena gracilis* (*CYP97H1*), have also been engineered into *E. coli*, *Y. lipolytica*, and *S. cerevisiae* (Tian et al., 2004; Yang et al., 2014; Cui et al., 2019; Tamaki et al., 2019; Wang et al., 2023; Soldat et al., 2024).

Thus, hydroxylation is the step that converts pigment backbones into market-relevant xanthophylls and expands the palette for downstream chemistry, as detailed in Figure 1e, which highlights the CrtZ/BCH- and CYP97-mediated conversions to zeaxanthin, lutein, and cryptoxanthin.

### 2.6 Ketolation reactions—astaxanthin formation

Ketocarotenoids (astaxanthin, canthaxanthin, echinenone) have vivid coloration and exceptional antioxidant properties, which are central to aquaculture pigmentation, cosmetic applications, and nutraceutical/pharmaceutical uses. Ketolation modifies the ionone rings of β-carotene, yielding ketocarotenoids (Tran and Kaldenhoff, 2020). Engineering efforts focus on β-carotene ketolases (e.g., *crtW* and BKT) and β-hydroxylases (crtZ) to divert flux toward astaxanthin (Martín et al., 2008; Allen et al., 2022; Abdullah et al., 2025; Hou et al., 2025).

Bacterial CrtW enzymes, especially from *Brevundimonas* sp., showed robust ketolation activities in *E. coli* and yeast, with mutagenesis boosting astaxanthin titers to 81 mg/L in 5-L bioreactors (Ye et al., 2006; Wang R. et al., 2017). Co-expression of *crtZ* and *crtW* enabled 1.82 g/L astaxanthin production in fed-batch fermentation (Zhang et al., 2022). Expressing bacterium *Pantoea agglomerans* β-carotene hydroxylases (*crtZ*) and alga *Haematococcus pluvialis* β-carotene ketolases (*HpBKT*) in *Synechocystis* sp. PCC 6803 converted β-carotene to astaxanthin via echinenone and canthaxanthin. Balanced expression of ketolases and hydroxylases, achieved by tuning promoter strength and expression ratios to harmonize their catalytic activities, was essential for efficient flux (Liang H. et al., 2023).

Taken together, precise tuning between hydroxylases and ketolases is the crux of industrial astaxanthin pathways, as shown in Figure 1f, where balanced CrtW/BKT and CrtZ activities channel β-carotene toward astaxanthin.

### 2.7 Epoxidation and isomerization reactions—violaxanthin and downstream carotenoids

The zeaxanthin–antheraxanthin–violaxanthin route links pigment composition with photoprotection. Engineering this node can modulate light stress responses. Epoxidation and de-epoxidation reactions form the xanthophyll cycle, crucial for balancing photoprotection and carotenoid flux in plants and algae. The epoxidation of zeaxanthin to violaxanthin, catalyzed by zeaxanthin epoxidase (ZEP), is a critical step in the xanthophyll cycle, essential for responding to light stress and maintaining a balance of carotenoids.

Zeaxanthin epoxidase (ZEP) converts zeaxanthin to violaxanthin. In the microalga *Nannochloropsis oceanica*, *NoZEP1/2* modulate violaxanthin pools and non-photochemical quenching (Liu et al., 2023). Expressing the alga *Ulva prolifera* ZEP in *S. cerevisiae* and *Chlamydomonas reinhardtii* improved salt tolerance, consistent with enhanced xanthophyll-cycle activity and increased violaxanthin formation (He et al., 2024). The engineering of violaxanthin de-epoxidase (VDE) remains limited, although strategies such as redox circuit optimization and balancing ZEP/VDE expression could be explored (Xinrui et al., 2023).

A review of violaxanthin biosynthesis and heterologous production in *E. coli* and yeast has been published (Takemura et al., 2021). Expressing bamboo VDE shifted the xanthophyll equilibrium toward zeaxanthin, lowering violaxanthin content, and enhanced quenching and stress resilience in *Arabidopsis* (Lou et al., 2022), while ZEP mutations (a single recessive splicing mutation) altered carotenoid balance in pepper (*Capsicum annuum*) (Lee et al., 2021). In *E. coli*, expressing the *crtY*, *crtZ*, and *ZEP genes* and optimizing redox balance resulted in violaxanthin production of 25.3 mg/g DCW (Xinrui et al., 2023). Expression of *Capsicum annuum* capsanthin/capsorubin synthase *CsCCS* yielded 6.77 mg/g capsanthin and 2.18 mg/g capsorubin from antheraxanthin and violaxanthin (Chen et al., 2025).

Hence, epoxidation engineering serves both physiology (light tolerance) and product diversification (epoxy- and keto-xanthophylls), as summarized in Figure 1g, which situates ZEP within the violaxanthin cycle.

### 2.8 Carotenoid cleavage enzymes in apocarotenoid biosynthesis

Cleavage defines the branching from pigments to signaling molecules. Carotenoid cleavage dioxygenases (CCDs) and 9-cis-epoxycarotenoid dioxygenases (NCEDs) generate apocarotenoids such as β-ionone and abscisic acid (ABA). CCDs and the specialized subfamily, NCEDs, are key enzymes in apocarotenoid biosynthesis, producing compounds like β-ionone, crocetin dialdehyde, and ABA. CCDs are non-heme iron-dependent enzymes that cleave carotenoid polyenes via a dioxygenase mechanism, forming keto or aldehyde cleavage products (Ahrazem et al., 2016; Wang et al., 2022). NCEDs, a CCD subfamily, specifically cleave 9-cis-epoxycarotenoids to xanthoxin—the direct ABA precursor—thereby linking carotenoid turnover to stress-responsive hormone biosynthesis (Daruwalla and Kiser, 2020). Overexpressing *Oryza sativa Os*NCED3 in *Arabidopsis* and rice increased ABA and stress tolerance (Hwang et al., 2010; Feng et al., 2024). Their catalytic mechanisms, active site architecture, and substrate specificity have been elucidated through structural and biochemical studies. CCD clade diversity and their bond-cleaving roles across carotenoids have been extensively reviewed (Harrison and Bugg, 2014; Ahrazem et al., 2016; Daruwalla and Kiser, 2020).

Plant CCD1 and CCD4 enzymes cleave β-carotene to produce β-ionone and other volatiles. *Dendrobium officinale* CCD1 forms β-ionone and pseudoionone from β-carotene and lycopene, respectively (Wang et al., 2022). Expressing *Nicotiana tabacum Nt*CCD1-3 in yeast, with a K38A mutation and redox balancing, boosted β-ionone yields (Gong et al., 2022). In *S. cerevisiae*, precursor pool size and membrane access were key bottlenecks (López et al., 2020). Microbial hosts, such as *Yarrowia lipolytica* and *Candida tropicalis*, have been engineered for β-ionone production, yielding 358 mg/L and 400.5 mg/L, respectively, in shake flasks through enhanced carotenogenic flux and CCD1 expression (Lu et al., 2020; Xu et al., 2024). Enhancements include membrane anchoring, NADPH/ferredoxin boosting, and modular cassette design. CCDs paired with enoate reductases also enable chemo-enzymatic conversion to dihydro-β-ionone (Qi et al., 2022).

Additionally, CCD4 can cleave zeaxanthin to crocetin dialdehyde, initiating crocin biosynthesis. *Gardenia jasminoides* CCD4a exhibited efficient crocin formation, with an expanded product profile that included cleavage of β-carotene, lycopene, and β-apo-8′-carotenal, yielding diverse C_17_–C_20_ dialdehyde apocarotenoids (Zheng et al., 2022). A recent CCD-independent route in *E. coli* using an engineered CrtMLIKE, CrtN, and CrtP enzymes to produce crocetin dialdehyde, where CrtM catalyzes head-to-head condensation of geranylpyrophosphate (GPP) to C20-phytoene; CrtN mediates sequential desaturation to form 8,8′-diapo-carotenoids; and CrtP performs further oxidation leading to crocetin dialdehyde. This strategy directly converted GPP to a C20 intermediate, thereby bypassing the multi-step β-carotene- and CCD-dependent route, reducing enzyme load and yielding 1.13 mg/L of crocetin dialdehyde. (Lee et al., 2025).

Beyond these metabolites, carotenoid cleavage also drives the biosynthesis of strigolactone (SL), a class of apocarotenoid-derived phytohormones with crucial developmental and ecological functions. SLs originate from the sequential cleavage of 9-cis-β-carotene by CCD7 and CCD8, yielding carlactone, which is further oxidized by cytochrome P450s of the MAX1 (CYP711) family into carlactonoic acid and various canonical and non-canonical SLs (Yoneyama et al., 2009; Alder et al., 2012; Jia et al., 2018). These compounds regulate shoot branching and root architecture and mediate rhizosphere signaling, promoting arbuscular mycorrhizal symbiosis but also triggering germination of parasitic weeds such as *Striga* and *Orobanche* (Ito et al., 2017; López-Ráez et al., 2017). Recent work has advanced microbial reconstruction of SL biosynthesis. A peach (*Prunus*) CYP711A homolog was shown to catalyze multi-step oxidation to strigol, the first identified SL, and the entire pathway from xylose feedstock to strigol was built in an *E. coli*–yeast consortium, proving the feasibility of industrial-scale SL production (Wu et al., 2023).

Collectively, cleavage chemistry extends the pathway beyond pigments into hormones, aromas, and specialty ingredients, as shown by the side of the modules in Figures 1c–f, marking the transition from carotenoids to apocarotenoids.

A consolidated overview of all engineering strategies, associated host systems, yields, and fold improvements across Section 2 is presented in Table 1.

**TABLE 1:** Summary of metabolic engineering strategies for carotenoid and apocarotenoid biosynthesis across hosts, core-pathways, and tailoring modules.

Host	Target product	Engineering strategy	Titer/yield	Fold improvement	References
Precursor Engineering (MEP, MVA, IUP)					
*S. cerevisiae*	Geraniol, α-humulene, squalene	Dual cytosol + peroxisome MVA targeting, relief of HmgR/Idi/Erg12 bottlenecks	—	Geraniol 94× α-humulene 60×, squalene 35×	Mukherjee et al. (2022)
*S. cerevisiae*	IPP/DMAPP, squalene β-carotene	MVA pathway retargeted to mitochondria	—	IPP/DMAPP 1.3×, squalene 2.8×, β-carotene 1.4×	Yanagibashi et al. (2024)
*C. reinhardtii*	Limonene	IUP + IDI + MsLS, 10 mM isoprenol, opt2 medium, 16:8 light–dark, 15 mL dodecane overlay	117 μg/L	117×	Zhao et al. (2022)
*E. coli*	Geraniol	Full MEP pathway integrated into genome + plasmid expressing ispA* (S80F) + *tObGES*	15 mg/L (36 h)	5× vs. plasmid system	Raghavan et al. (2024)
*Z. mobilis*	Isoprene	Complete flux balancing (upstream + downstream) + carbon sink	33.3 nmol/mmol glucose	5.9×	Khana et al. (2023)
*S. cerevisiae*	Multiple terpenoids (limonene, amorphadiene, taxadiene β-amyrin, lycopene)	Combined IUP + native MVA to build a universal IPP/DMAPP platform	—	Limonene 27×, amorphadiene 2.7×, taxadiene 5.6× β-amyrin 3×, lycopene 2×	Ma et al. (2023)
*E. coli*	Lycopene β-carotene, linalool	Best IK/IPK pair (SmDAGK + MvIPK) + optimized lycopene operon (LYC4) + high-activity CrtYB for β-carotene + GPPS + LS module for linalool + two-stage fermentation + substrate optimization	Lycopene 300 mg/L, β-carotene 248 mg/L, linalool 364 mg/L	Lycopene 4–6× β-carotene 3×, linalool 2.6×	Ma X. et al. (2022)
GA02 (*E. coli* MG1655 ΔrecA ΔendA DE3)	Geranate	Optimized IUP (EcthiM + MvIPK + EcIDI) + geraniol pathway (AgGPPS + ObGES) + controlled CdGeDH/CdGaDH expression on low-copy pSC101 under PcymO, reducing by-products	764 mg/L	2.6× vs. GA01 (298 mg/L)	Pan et al. (2023)
*S. cerevisiae*	Squalene, limonene β-carotene	Replacement of MVA pathway with IU pathway, construction of IUPD chassis, growth-coupled ATP upregulation, directed evolution of SmDAGK S47A/L124A and AtIPK S270P/A272R, multi-copy IUP integration (IUP6, IUP8, IUP10), boosted IPP/DMAPP, GPP, FPP, GGPP supply	Squalene 26.02 mg/g/OD, limonene 3.42 mg/g/OD, β-carotene 1.86 mg/g/OD	Squalene 695×, limonene 850× β-carotene 18×	Li et al. (2024b)
Initial condensation steps (GGPP and Phytoene Synthesis)					
*Y. lipolytica*	Zeaxanthin	Construction of the lycopene pathway (*crtE, crtB, crtI*), then β-carotene pathway (*carRP*), screening of three CrtZ enzymes, selection of *Eu-crtZ* (*Pantoea ananatis*), multi-copy integration into rDNA, TEF1-driven high expression	21.98 mg/L zeaxanthin (YPD, 5 days)	4.02×	(Xie et al., 2021)s
*A. thaliana*	Total carotenoids (phytoene → α/β-carotene derivatives)	Chimeric PSY–GGPS11 fusion (PYGG) enabling direct GGPP channeling to the carotenoid pathway, bypassing competition with chlorophyll, tocopherol, and gibberellin branches	Up to 3000 μg/g DW in callus	1.5× vs. high-PSY lines, >2× vs. WT	Camagna et al. (2019)
*A. thaliana* (GGPPS5/8/9 lines)	Carotenoids	Overexpression of LtuGGPPS2 from *Liriodendron tulipifera*, plastid-localized GGPP enhancement, upregulation of MEP-pathway genes (*dxr, hds, hdr, AtGGPPS, AtGPPS*), increased GGPP supply	Carotenoids ↑39.24%	1.39×	Zhang et al. (2021)
*Solanum lycopersicum* (tomato)	Lycopene	Fruit-specific overexpression of EutPSY (Elaeagnus umbellata PSY) under the E8 promoter, upregulation of SlLCYb and SlCYCB, downregulation of SlLCYe, enhanced carotenoid flux	—	1.7–2.6× carotenoids	Wang et al. (2020)
*Dunaliella salina*	Carotenoids	Overexpression of DsORHis (R129H Orange protein mutant) to enhance interaction with DsPSY1/2, stabilization of PSY, enlarged plastoglobuli and plastid differentiation, increased carotenoid storage capacity	Carotenoids ↑73.4% vs. WT	1.73×	Liang et al. (2023b)
*Haloferax volcanii*	Bacterioruberin	Overexpression of HVO-PSY (phytoene synthase) to increase GGPP→phytoene flux, optimization of culture (150 g/L NaCl, 45 °C, pH 6.5–7.5)	773 μg/g DCW	1.38×	Lin et al. (2025)
*E. coli* BL21 (DE3)	β-carotene	Expression of Pyropia yezoensis PSY (PyPSY) with *crtE/crtI/crtY* to validate PSY functionality by pigment complementation	—	Functional restoration from 0 to detectable β-carotene	Li et al. (2024a)
Desaturation and isomerization (Phytoene to Lycopene Conversion)					
*S. cerevisiae*	Lycopene	Multi-modular engineering: (1) acetyl-CoA enhancement via SeACS1 (with ΔMLS1), (2) strengthened MVA pathway (ERG10, ERG12, ERG20, tHMG1), (3) optimized carotenoid module (PaCrtE, PagCrtB, BtCrtIs), (4) ERG9 downregulation via GRE1, (5) increased NADPH via POS5, (6) efflux engineering via PDR11	343.7 mg/L	4.3×	Huang et al. (2024)
*S. cerevisiae*	Lycopene	Multi-layer metabolic and enzyme engineering: deletion of DPP1/LPP1 to block farnesol, ERG9 UAS deletion to reduce sterol flux, deletion of ROX1/MOT3 to derepress MVA, directed-evolution mutants CrtEMB2 and CrtBM1, δ-integration for multi-copy insertion, OLE1-mediated membrane flexibility, STB5-driven NADPH enhancement	41.8 mg/g DCW	74.6×	Hong et al. (2019)
*E. coli*	Lycopene	Highly active *P. agglomerans* crtE/crtB/crtI, addition of mevalonate bottom pathway (*mvaK1, mvaK2, mvaD, idi*) to boost IPP/DMAPP, no IPTG induction to avoid toxicity and optimize flux	60 mg/L (48 h, 2 YT, glycerol, mevalonate)	2.2×	Yoon et al. (2007)
*Nicotiana tabacum*	Carotenoids	Overexpression of wolfberry LcCRTISO (carotene cis–trans isomerase) compared with LcPDS and LcZDS, CRTISO was identified as a key desaturation-pathway regulator for carotenoid flux	—	2.4×	Li et al. (2020)
*E. coli*	β-carotene	Complete reconstruction of *D. salina* β-carotene pathway (PDS–ZISO–ZDS–CRTISO) in *E. coli*, validation of ZISO/CRTISO essentiality, discovery of 7,7′,9,9′-tetra-cis-β-carotene	3.3 mg/g DCW	1.2×	Chen et al. (2023)
Cyclization reactions (formation of α- and β-carotene)					
*Y. lipolytica*	β-carotene	Removal of lycopene cyclase substrate inhibition via CarRP(Y27R), balanced flux via GGPPS-mediated flow restriction, strengthened MVA (tHMGR, ERG12, IDI, ERG20), introduction of synthetic IUP (CK + IPK), optimized C/N ratio to support lipid-based carotenoid sequestration	39.5 g/L (3-L fed-batch, 494 mg/g DCW, 0.165 g/L/h)	1,441× vs. initial strain	Ma Y. et al. (2022)
*Chlamydomonas reinhardtii*	β-carotene	Nuclear genome integration and expression of bacterial CrtY (from *P. agglomerans*) under psaD promoter, codon optimization, chloroplast transit peptide for plastid targeting, high-light induction to stimulate carotenoid biosynthesis	30.65 mg/g DW	2.45×	Huang et al. (2023)
*E. coli*	α-carotene (major) β-carotene (second major)	Co-expression of DbLcyB (bifunctional β-/ε-cyclase) and DbLcyE (ε-/β-monocyclase) from *Dunaliella bardawil* with lycopene-producing pAC-LYC plasmid to reconstruct α- and β-ionone ring formation	55% α-carotene, 42% β-carotene (balance <3% other carotenoids)	Product ratio study, no fold-change	Liang et al. (2019)
*Ipomoea batatas*	Total carotenoids β-carotene	Overexpression of IbLCYB2 (lycopene β-cyclase from sweetpotato HVB-3) enhanced carotenoid branch flux, carotenoid globule formation, and upregulation of carotenoid and ABA-biosynthesis genes	—	Total carotenoids 2.57×, β-carotene 3.41×	Kang et al. (2018)
*Trichoderma reesei*	β-carotene	Multi-copy integration of *carRP* (phytoene synthase/lycopene cyclase) and *carB* (phytoene dehydrogenase), MVA strengthening via Trhmg1 and XdCrtE, medium optimization (10 g/L tryptone, 80 g/L glucose)	286.63 mg/L (30-L fed-batch, 6.31 mg/g DCW)	29.1× vs. MuQ1 (9.85 → 286.63 mg/L)	Li et al. (2023)
*C. reinhardtii*	β-carotene	Nuclear expression of codon-optimized bacterial CrtY (from Pantoea agglomerans) fused to a chloroplast transit peptide, driven by psaD promoter, genomic integration, and light-induced carotenoid stimulation	30.65 mg/g DW	(2.45x)	Huang et al. (2023)
Hydroxylation reactions (zeaxanthin production)					
*Y. lipolytica*	Zeaxanthin	carB → carRP chassis to generate β-carotene, pathway strengthening via ERG12 and ERG20, flux channeling with IDI–GGS1 and ERG20–GGS1 assemblies, MVA boosting (*mvaE, mvaSMT*), β-carotene hydroxylation by *crtZ*, cofactor enhancement via RFNR1, additional *crtZ* and *carRP* copies	775.3 mg/L	39×	Zhang et al. (2023)
*Y. lipolytica*	Zeaxanthin	Expression of PaCrtZ as core β-carotene hydroxylase, SKL peroxisome-targeting tag to match β-carotene localization, improved access to stored β-carotene, and increased catalytic conversion	412 mg/L	1.66×	Soldat et al. (2024)
Ketolation reactions (astaxanthin and other ketocarotenoids)					
*E. coli* MG1655	Astaxanthin	Error-prone PCR and mutant screening of CrtW to identify CrtW (L175M) with enhanced ketolation, improved adonixanthin → astaxanthin conversion, reduced intermediate accumulation	78% astaxanthin (product fraction)	1.47×	Ye et al. (2006)
*S. cerevisiae*	Astaxanthin	Screening CrtZ/CrtW pairs, identification of AspCrtZ + BDC263CrtW as optimal, increasing CrtZ via promoter swap (FBA1p→TEF1p), carbon-restricted fed-batch to maximize flux and biomass	81.0 mg/L (5-L bioreactor)	2.4×	Wang R. et al. (2017)
*E. coli*	Astaxanthin	Establishment of high-flux β-carotene→canthaxanthin→adonirubin chassis (CAR026/Can004), characterization of PAcrtZ (β-carotene→zeaxanthin) and *PCcrtZ* (canthaxanthin→astaxanthin), use of two *PAcrtZ* + one *PCcrtZ* copy, chromosomal integration of crtZ/PCcrtZ/PCcrtZ–crtW*, pH 7.0 fed-batch	1.82 g/L (5-L fed-batch)	1.65×	Zhang et al. (2022)
*Synechocystis* sp	Astaxanthin (trace), canthaxanthin (major)	Silencing endogenous CrtO to eliminate β-carotene→echinenone, introduction of HpBKT (β-carotene ketolase), PaCrtZ expression to convert canthaxanthin→astaxanthin, LC–MS/MS confirmation of pathway, astaxanthin detected as minor product	Astaxanthin minor, canthaxanthin major	Pathway elucidation, no titer	Liang et al. (2023a)
*Chlamydomonas reinhardtii*	Canthaxanthin	Overexpression of native CrBKT under HSP70A/RBCS2 promoter, screening for DARK-PALE phenotypes under heterotrophic dark culture, reduced chlorophyll/carotenoid ratio enabling ketocarotenoid detection, CrBKT is active only in dark-grown transformants	10% of total carotenoids	No WT ketocarotenoids, no mg/L	Tran and Kaldenhoff (2020)
*Nicotiana benthamiana*	Astaxanthin	Introduction of Adonis CBFD2→HBFD1 pathway, plastid-targeted expression from bidirectional 35S/FMV promoters, coordinated three-step β-ring dehydrogenation + hydroxylation of β-carotene, efficient astaxanthin formation with minimal canthaxanthin	0.99 mg/g DW	2.5–7.5× vs. crtW or Cit/crtW lines	Allen et al. (2022)
*Y. lipolytica*	Astaxanthin	Construction of β-carotene chassis (CarRPY27R→CarB), MVA boosting (tHMG1, GGPPSa, MvaS/MvaE, IDI, Erg20MTF88S), NADPH increase (Zwf1, Gnd1), modular HpCrtZ-RIAD/HpBKT-RIDD assemblies, directed evolution (HpBKT-V264D, HpCrtZ-N183A/Y219H), multi-copy 26S rDNA integration, lipid-body relocalization, LEU2/URA3 restoration, DMSO-enhanced membrane fluidity	2.82 g/L (5-L, 2752 mg/L intracellular +68 mg/L extracellular)	229×	Abdullah et al. (2025)
Epoxidation reactions (violaxanthin, capsanthin, capsorubin)					
*E. coli*	Violaxanthin	Genome-integrated MVA pathway, *crtEBI* lycopene chassis, promoter-library balancing of *crtY/crtZ* (DZ12 chassis), optimized Capsicum annuum ZEP on low-copy pSC101, redox engineering via ΔtrxB, co-expression of CtFd/CtFNR electron-transfer chain, minimized zeaxanthin/β-carotene/antheraxanthin byproducts	25.28 mg/g DCW	60×	Xinrui et al. (2023)
*Nannochloropsis oceanica*	Violaxanthin and related xanthophylls	Overexpression of NoZEP1 to enhance zeaxanthin epoxidation, increased violaxanthin, vaucheriaxanthin, neoxanthin, higher total carotenoids, and chlorophyll a under high light	40%–60% carotenoid increase under high light	Phenotypic, no absolute titer	Liu et al. (2023)
*Chlamydomonas reinhardtii*	Violaxanthin-cycle xanthophylls	Heterologous expression of UpZEP, increased zeaxanthin epoxidation, higher violaxanthin/antheraxanthin under salt stress, improved chlorophyll retention, and biomass at 120 mM NaCl	Violaxanthin, lutein, and zeaxanthin increased	Phenotypic enhancement, no titer	He et al. (2024)
*Capsicum annuum*	Zeaxanthin, total carotenoids	Natural ZEP splice-site mutation causing a truncated protein, impaired zeaxanthin epoxidation, zeaxanthin accumulation to 60% of carotenoids, increased total carotenoids, and altered chromoplast ultrastructure	56.49 mg/100 g FW (orange) vs. 16.73 mg/100 g (yellow)	3.4× total carotenoids, substantial zeaxanthin gain	Lee et al. (2021)
*E. coli*	Capsanthin, capsorubin	Chloroplast-like redox (ΔtrxB + Trx-m1/m2/m4) to activate ZEP, Cpn60α/β + Cpn20 chaperones to fold CCS, SpyTag/SpyCatcher-based CCS homotrimers, β-cav1-mediated membrane localization, FDHm + Fre FADH_2_ regeneration, 20 mM formate feeding	Capsanthin 6.77 mg/g DCW, capsorubin 2.18 mg/g DCW	capsanthin 24.18×	Chen et al. (2025)
Cleavage reactions — apocarotenoids (β-ionone, crocins, ABA, strigol)					
*E. coli*	β-ionone	Heterologous expression of DoCCD1, β-carotene cleavage at 9,10/9′,10′, strong β-ionone formation by GC–MS, complete loss of carotenoid pigmentation	Qualitative GC–MS peak	Functional assay, no WT baseline	Wang et al. (2022)
*S. cerevisiae*	β-ionone	Markerless CRISPR/Cas9 integration of CrtE→CrtYB→CrtI modules into XI-5→XI-3→X-2 loci, β-carotene increased 4→32 mg/g DCW, MVA boosting via two extra tHMG1 copies, integration of engineered fyn-PhCCD1 (membrane-targeted), stepwise CCD1 copy increase (β-iono5.1/5.2/5.3)	33 mg/L (shake flask)	19×	López et al. (2020)
*Y. lipolytica*	β-ionone	Module 3 (carB→carRP→PhCCD1) on high β-carotene chassis, Module 2 amplification (tHMG1, GGS1, ERG10/ERG13, ERG8/ERG12/ERG19, IDI/ERG20), acetyl-CoA boosting via bbPK + bsPTA, extra Module 3 copy, nitrogen optimization, fed-batch with 15% DO control	0.98 g/L (3-L bioreactor)	280×	Lu et al. (2020)
*Candida tropicalis*	β-ionone	Compartment engineering (peroxisomes→cytosol→lipid droplets), CCD1 screening (PhCCD1 best), directed evolution (PhCCD1-C61, PhCCD1-sE13), co-expression of mutants in peroxisomes, cytoplasm, LDs, multi-copy integration, two-phase dodecane system, DO-controlled fed-batch	730 mg/L (5-L, 11.8 mg/g DCW, 320 h)	5.8×	Xu et al. (2024)
*S. cerevisiae*	β-ionone	High-activity NtCCD1-3 screening, multi-copy δ-integration, K38A membrane-binding mutant, NADPH supply boosted via POS5, dodecane overlay for *in situ* extraction	1.08 mg/L	3.1×	Gong et al. (2022)
Cell-free enzymatic system	Dihydro-β-ionone	PhCCD1 screening for β-apo-8′-carotenal cleavage, coupling with AaDBR1 enoate reductase for β-ionone→dihydro-β-ionone, enzyme ratio and pH 8.0/45 °C optimization, NADPH regeneration via GDH + glucose	13.34 mg/L (3 h, 85.8% molar conversion)	5.3×	Qi et al. (2022)
*N. benthamiana*	Crocins (I & II)	Overexpression of GjCCD4a without additional pathway genes, endogenous oxidases convert crocetin dialdehyde, endogenous UGTs glycosylate crocetin, resulting in strong crocin accumulation in leaves	1.61 mg/g DW total crocins (I & II)	2.4×	Zheng et al. (2022)
*E. coli*	Crocetin dialdehyde	CrtMLIKE active-site engineering (V133F/V237W) to enforce GPP–GPP C20 condensation, C30 desaturase CrtN plus oxidase CrtP for CCD-free apocarotenoid pathway from GPP, first oxidative-cleavage-free crocetin dialdehyde route	1.13 mg/L	4.5× lower than CCD2-based 5.14 mg/L (proof-of-concept)	Lee et al. (2025)
*Oryza sativa*	Abscisic acid (ABA)	OsNCED3 overexpression to raise ABA to +50.9%, activation of ABA-responsive genes (*OsSalT, OsWsi18*), improved osmotic adjustment (↑proline, soluble sugar, starch), better ion homeostasis (↑K^+^/Na^+^, Ca^2+^/Na^+^), reduced ROS/membrane injury, enhanced antioxidant enzymes	—	5.5×	Feng et al. (2024)
*E. coli–S. cerevisiae* consortium	Strigol (strigolactone)	Screening peach MAX1 paralogs, identification of PpMAX1c (Group III MAX1), co-expression with ATR1 in *S. cerevisiae*, co-culture with carlactone-producing *E. coli*, multi-step CL→CLA→18-OH-CLA→strigol oxidation	71.82 μg/L	Functional gain vs. PpMAX1a/b (no strigol)	Wu et al. (2023)

Abbreviations: MEP, methylerythritol phosphate pathway; MVA, mevalonate pathway; IUP, isopentenol utilization pathway; IPP, isopentenyl pyrophosphate; DMAPP, dimethylallyl pyrophosphate; GPP/FPP/GGPP, geranyl-/farnesyl-/geranylgeranyl-pyrophosphate; DCW, dry cell weight; DW/FW, dry/fresh weight; PSY/CrtB, phytoene synthase; CrtE/GGPPS, geranylgeranyl pyrophosphate synthase; CrtI, phytoene desaturase; PDS/ZISO/ZDS/CRTISO, plant-type desaturases/isomerases; LCYB/LCYE/CrtY, β/ε-cyclases; CrtZ, β-carotene hydroxylase; CrtW/BKT, β-carotene ketolase; ZEP, zeaxanthin epoxidase; CCS, capsanthin–capsorubin synthase; CCD1/CCD4, carotenoid cleavage dioxygenases; NCED, 9-cis-epoxycarotenoid dioxygenase; MAX1, strigolactone-biosynthesis P450; CL, carlactone; CLA, carlactonoic acid; 18-OH-CLA, 18-hydroxy-carlactonoic acid; SLs, strigolactones; TEF1p/FBA1p, constitutive yeast promoters; psaD, *chlamydomonas* nuclear promoter; HSP70A/RBCS2, algal hybrid promoter; SKL, peroxisomal targeting signal; δ-integration, multi-copy chromosomal integration; DO, dissolved oxygen; Two-phase extraction, dodecane overlay for *in situ* capture; Flux balancing, coordinated precursor/pathway tuning; ATP-growth coupling, engineered linkage of metabolite production to growth.

## 3 Discussion

The engineering advances outlined above and summarized in Table 1 reveal how each biosynthetic module contributes to the overall efficiency and diversity of carotenoid and apocarotenoid production. The discussion that follows synthesizes these developments to highlight common design principles, challenges, and opportunities.

### 3.1 Current challenges in microbial carotenoid and apocarotenoid biosynthesis

The challenges limiting predictable, scalable carotenoid and apocarotenoid production stem from several interconnected biological constraints. Precursor limitations and imbalanced fluxes remain central issues, as even strengthened MVA and MEP pathways exhibit fluctuations tightly coupled to growth rate and cellular redox balance (Kang et al., 2016; Zhao et al., 2022; Raghavan et al., 2024). Redox imbalance caused by demanding enzymes, such as CrtI, can deplete reducing equivalents (NADPH/NADH) during sequential desaturation steps, introducing flux bottlenecks and metabolic stress. Regulatory limitations further constrain pathway performance because most systems rely on constitutive promoters; however, carotenoid synthesis requires dynamic control to prevent metabolic burden. Product specificity is strongly influenced by the balance of cyclases and tailoring enzymes LCYB/LCYE, CrtZ, BKT/CrtW, and ZEP, whose optimization is complicated by cofactor requirements and substrate competition. Spatial factors add another challenge, as unstable intermediates would diffuse or degrade without appropriate compartmentalization. Additionally, the field remains heavily centered on *S. cerevisiae* and *E. coli*, leaving algae, fungi, and other bacterial hosts underexplored despite their unique advantages. Finally, industrial implementation demands solutions for scale-up, especially low-cost feedstocks, robust high-stress sustaining strains, and efficient downstream processing tailored to highly hydrophobic carotenoids.

### 3.2 Strategies emerging from recent engineering advances

The engineering concepts discussed in Section 2 address the interconnected challenges above. Precursor balancing and redox management remain foundational. Strengthened MVA and MEP pathways increase IPP/DMAPP availability. However, optimal flux requires redox-balanced pathway design, the use of feedback-resistant enzyme variants, and machine-learning-guided promoter tuning. AlphaFold-enabled structural models now support rational redesign of PSY, a longstanding catalytic bottleneck.

Modular desaturation and isomerization strategies combine the robustness of bacterial CrtI with the regulatory precision of plant desaturation complexes (Schaub et al., 2012; Li et al., 2020). Dynamic control using inducible, quorum-sensing, or metabolite-responsive promoters (Wan et al., 2019; Stephens and Bentley, 2020) enables tunable desaturation while preventing redox collapse.

Cyclization precision is strongly governed by the LCYB/LCYE ratio, which determines the ratio of α-to β-carotene formation (Kang et al., 2018; Liang et al., 2019). Directed evolution and CRISPR-based genome editing provide powerful means for modulating cyclase specificity, especially in hosts with existing carotenoid machinery.

Hydroxylation, ketolation, and epoxidation efficiencies depend heavily on tailoring enzymes such as CrtZ, BKT/CrtW, and CYP97 homologs, whose activities remain constrained by cofactor requirements and kinetic limitations (Zhang et al., 2022; Lautier et al., 2023). ZEP engineering is still in its early stages, which requires improving catalytic efficiency and electron-transfer support (Liu et al., 2023; He et al., 2024).

Spatial engineering strategies address the instability of intermediates by leveraging organelle targeting, peroxisome engineering (Choi et al., 2022), lipid droplet remodeling (Lin et al., 2024), scaffold-based assemblies (Wang Y. et al., 2017; Kang et al., 2019), and emerging synthetic bacterial microcompartments (Yung, 2015; Moon et al., 2024) that enhance colocalization.

Tailoring apocarotenoid enzymes such as CCD1 and NCED unlocks higher-value apocarotenoids, including β-ionone and ABA. Specialized CCDs, such as CCD2, catalyze the formation of crocetin dialdehyde, a key intermediate in saffron crocins (López et al., 2020; Chen et al., 2025). Future opportunities include extremophile enzyme mining, intermediate stabilization, and engineered microbial consortia to distribute pathway burden.

### 3.3 Future perspectives

Drawing from these challenges and strategies, several future directions emerge. First, comprehensive kinetic and structural enzyme characterization remains essential, particularly for PSY, CRTISO, ε-hydroxylases, ZEP, and NCEDs, which represent key catalytic nodes that currently constrain carotenoid bioengineering. Second, system-level flux design integrating genome-scale metabolic models, proteomics, and AI-guided optimization will improve our ability to predict pathway performance and design balanced flux distributions that respond dynamically to metabolic demands (Blazeck and Alper, 2010; Mukherjee et al., 2022; Zhang and Wang, 2025). Third, diversification of host chassis, including algae, cyanobacteria, and non-model yeasts, offers untapped advantages in compartmentation, redox environment, and tolerance to metabolic stress (Santos-Merino et al., 2019). Fourth, dynamic pathway regulation using CRISPRi/a, optogenetics, and synthetic feedback loops presents a frontier for controlling flux, stress responses, and metabolite toxicity with temporal precision (Han et al., 2023). Finally, industrial translation depends on innovations in low-cost feedstocks, co-product strategies, strain robustness, and scalable downstream processing tailored to carotenoids and apocarotenoids (Su et al., 2023). Collectively, carotenoid and apocarotenoid engineering are moving towards precision synthetic biology, bridging enzyme discovery with rational design, expanding into novel hosts, and transforming proof-of-concept pathways into robust platforms for nutrition, pharmaceuticals, and sustainable agriculture.
